# Predicting fine-scale downstream migratory movement of Atlantic salmon smolt (*Salmo salar*) in front of a hydropower plant

**DOI:** 10.1038/s41598-024-80972-4

**Published:** 2024-12-28

**Authors:** Kim M. Bærum, Ana T. Silva, Henrik Baktoft, Karl Ø. Gjelland, Finn Økland, Torbjørn Forseth

**Affiliations:** 1 Norwegian Institute for Nature Research, Fakkelgården, 2624 Lillehammer, Norway; 2 Norwegian Institute for Nature Research, Postbox 5685, 7485 Trondheim, Norway; 3 National Institute of Aquatic Resources, Technical University of Denmark (DTU Aqua), 8600 Silkeborg, Denmark; 4 Fram Centre, Norwegian Institute for Nature Research, Langnes, PO Box 6606, 9296 Tromsø, Norway

**Keywords:** Individual based models, Spatial modeling, Fish behavior, Boosted regression trees, 3D telemetry, Fish conservation, Animal migration, Hydrology

## Abstract

The Atlantic salmon (*Salmo salar*) is an iconic species of significant ecological and economic importance. Their downstream migration as smolts represents a critical life-history stage that exposes them to numerous challenges, including passage through hydropower plants. Understanding and predicting fine-scale movement patterns of smolts near hydropower plants is therefore essential for adaptive and effective management and conservation of this species. We present a spatially explicit individual-based model for predicting the movement of Atlantic salmon smolts in regulated rivers in Norway, parameterised for smolt movements in the River Mandal and the River Orkla. The model is rooted in statistically derived relationships between observed smolt swimming behaviour and the hydraulic variables they encounter. The aim of the model was to provide fast yet representative swimming patterns past hydropower plants, based on the hydraulic conditions experienced by the smolts. The model outperformed a ‘drift-only’ model in portraying observed swim tracks when comparing simulated and observed tracks. It was found to represent smolt swimming behaviour well. Our results show that by constructing swim models using relatively simple and general statistical relationships between smolt swimming behaviour and the hydraulic environment, we can produce fast and relevant outputs for an adaptive management process, aimed at exploring how physical implementations or changes in flow regulations might affect smolt populations.

## Introduction

The urgent global need to transition towards renewable energy has underscored the importance of alternative sources such as hydropower^1^. Yet, hydropower operations have the potential to significantly impact biodiversity and ecosystem function in aquatic ecosystems^2^. Hydropower plants alter the natural flow of rivers, resulting in a series of harmful concomitant effects, including the loss of critical habitats for aquatic species, homogenization of habitats, changes in sediment transport dynamics, alterations in water temperature regimes, and disruption of migratory pathways and connectivity e.g.,^3–5^. These ecological consequences, especially concerning the free movement of fish, are of paramount concern in the context of sustainable hydropower development.

Migratory fish, such as Atlantic salmon, are vital components of aquatic ecosystems and often serve as indicators for conservation efforts due to their ecological, economical, and societal importance^6,7^. Ensuring the continued survival and propagation of Atlantic salmon requires addressing the challenges posed by hydropower infrastructure, as it can impede their migration, and affect the population dynamics. Indeed, hydropower increase the mortality rate during the downward migration as the smolts commonly follow the high velocities of the main flow at the power station, towards and into the turbines e.g.,^8,9^. Hydropower infrastructure may also increase mortality rates due to increased predation^10^. Besides the obvious direct negative effect of increased mortality, a more indirect effect of altered selection pressure for different behaviors can also arise, potentially altering the behavioral adaptability within a population^11^. To mitigate these negative and potentially lethal effect of hydropower operations, it is important to understand small-scale downstream migratory movements, and how these movement patterns are expected to change under different flow regimes^12,13^. This is vital knowledge for the formulation, and adaptive evaluation, of management strategies that may better balance the ecological effects against water resource practices.

In general, models for fish movement in response to their environment can range from fine scale and complex e.g.,^14,15^ to more broad scale patterns for a whole fish community e.g.,^16^ in how they incorporate movement decisions. The range of existing model frameworks, where most are tailored to a specific applied goal, also portray the difficulty of building movement models that adequately predict fish movements for multiple types of systems and management problems. For downstream movement of juvenile fish, the most commonly used model framework is individual based models (IBM) and are based on the mechanistic understanding of movement of fish and/or particles in the flow direction, where the rules of movement ranges from passive drift to more complex biased correlated random walks^14,17,18^. The more complex models usually describe observed smolt movement better e.g.,^19^, but are naturally also more computationally demanding both in time and computational power and might thus not be a feasible choice for exploring multiple potential mitigation actions against each other.

By analyzing small scale swimming behavior in the wild as functions of the hydraulic variables the smolt experiences (i.e., velocities and turbulence kinetic energy), consistent general patterns of swimming behavior (swimming direction and swimming speed) have been observed across different streams and populations of Atlantic salmon^13,20^. This suggests that not only do the smolts actively swim along the main flow during their migration^21^, they also choose diverging paths to the flow. The different behaviors are guided by the hydraulic environment^13^. Patterns of active swim behavior and avoidance behavior are also observed in smolts of Pacific salmon^22,23^. Thus, models predicting small scale downstream migration patterns of smolts past hydropower installations based on drift alone might not give a representative picture of actual smolt movement.

In this study, we develop a fine-scale individual-based movement model for the downstream migration of Atlantic salmon smolt, based on derived probabilities of different swimming behaviours as a function of hydraulic variables. Correlative relationship models generate outputs and predictions relatively quickly, are easy to interpret, and provide insights into general patterns across various situations. These relationships thus form a foundation for building more general models for small-scale swimming behaviour as functions of the hydraulic environment for Atlantic salmon smolts, enabling meaningful predictions across different systems and flow regimes. We evaluate the predicted smolt tracks from the swimming behaviour model against those from a drift model and observed smolt tracks. The model aims to balance simplicity with realism to predict representative swimming behaviour, which, for example, could be used to estimate expected mortality rates (e.g., smolts swimming into the turbine versus past the intake) under different flow conditions. Such predictions can inform various management considerations.

## Material and methods

### Biological and hydraulic data

In this study we used 2 D and 3 D biotelemetry data from 140 Atlantic salmon smolts (mean Lt: 14.70 ± 1.05 SD cm; mean W: 23.93 ± 5.91 SD g) collected in the vicinity of the intake of the Laudal HPP (Mandal river, 66 individuals^13^), and the Bjørset dam (Orkla river, 74 individuals^20^), Norway. Fish positions were estimated using YAPS^24^ resulting in a total of 32,994 positions used for this analysis. Fish were tagged with acoustic tags (Lotek M-626, burst interval 5 s, signal definition file 1.1.1) implanted in the peritoneal cavity. All handling and tagging procedures were performed according to the Norwegian regulations for treatment and welfare of animals and the experimental protocol was approved by the Norwegian Food Safety Authority (permit ID 7636). Throughout the study the authors also complied with the ARRIVE guidelines^25^. A total of fourteen and twenty-seven hydrophones (Lotek 200 kHz WHS 3050, Lotek Wireless Inc., Newmarket, Ontario, Canada) were positioned in the main water course and intake area of the Laudal hydropower weir in the Mandal river and at the intake area of the Svorkmo HPP, Orkla river; respectively. Further details on the telemetry studies can be found in Silva et al.^13^ and Szabo-Meszaros et al.^20^. The smolt-data was combined with hydraulic data collected by using of an RiverSurveyor S9 (Sontek) Acoustic Doppler Current Profiler (ADCP) and modeled using 3-dimensional computational fluid dynamics (CFD) model SSIIM (Sediment Simulation In Intakes with Multiblock option^26^), in the case of Mandal river, and CFD model OpenFOAM (release 4.1.0) in the Orkla river.

**Fig. 1:**
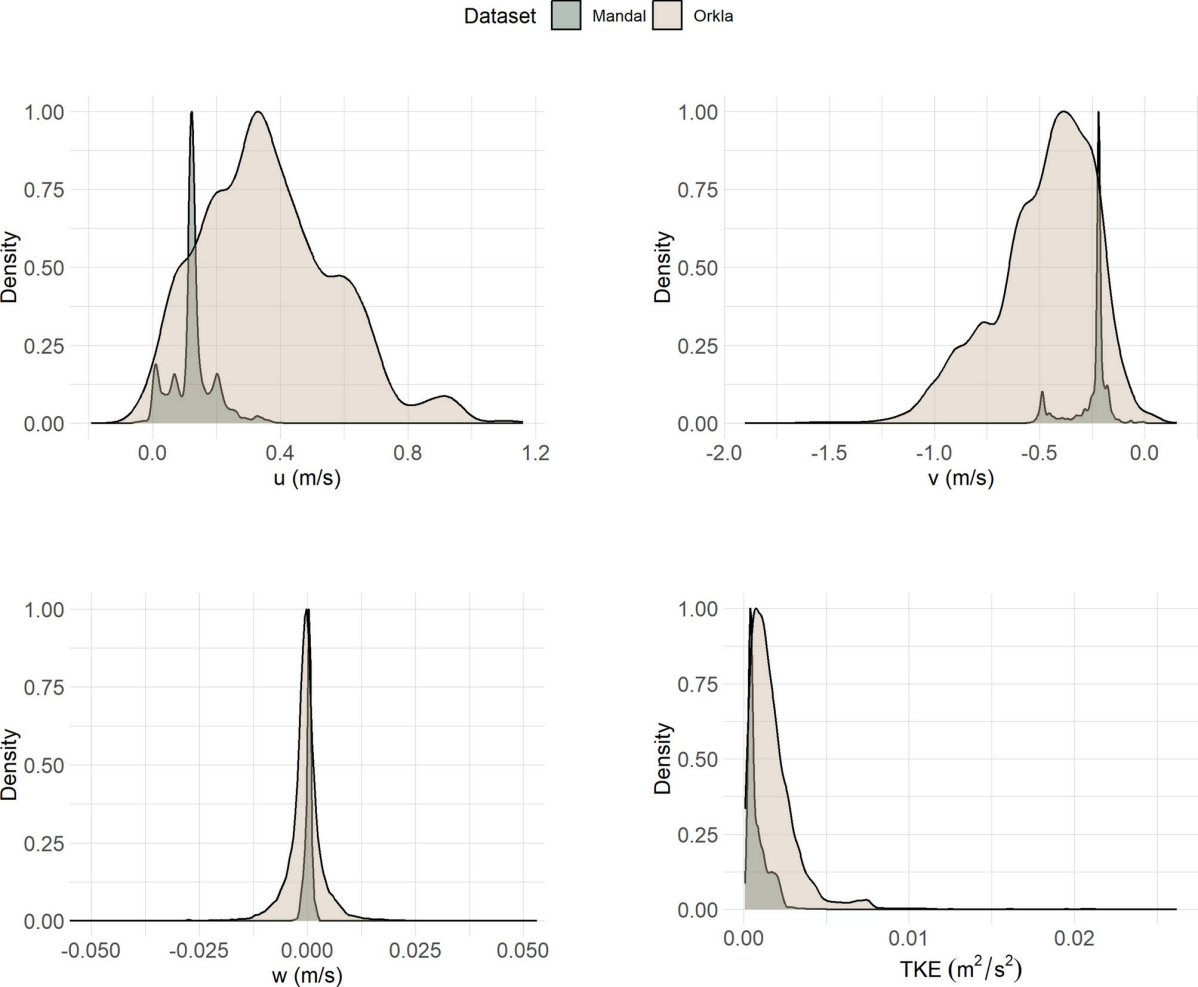
Density plots for the flow velocities (u, v and w) and turbulent kinetic energy (TKE) the tagged smolts encountered in Mandal (grey) and Orkla (light pink).

At the Mandal River system, four different hydraulic scenarios were simulated, each varying in total flow discharge. (Q = 82, 92, 94, and 98 m^3^ s⁻^1^) and respective percentages of flow directed into the bypass (PerQ = 64%, 89%, 49%, 44%). These conditions were selected as they were representative of the flow conditions encountered by smolts during the study period. At the Orkla river 10 scenarios were modelled to represent flow conditions encountered by the smolts (with Q varying between 47 and 211 m^3^ s⁻^1^). CFD modeling was used in this study to analyze the three-dimensional velocity components (longitudinal: u^+^ downstream, u^−^ upstream; transversal: v^+^ right to left bank, v^−^ left to right bank; vertical: w^+^ upwards, w^−^ downwards), and turbulent kinetic energy (TKE). The smolts in Orkla experienced in general a wider range of values for the three-dimensional velocity components and TKE compared to the smolts in Mandal (i.e., higher velocities, see Fig. 1) during their migration through the study areas. More details on catching, tagging, surgery, telemetry procedures and CFD modelling can be found in Silva et al.^13^ and Szabo-Meszaros et al.^20^.

### Statistical analysis

#### Simulating smolt movement

In this study, we used an individual-based model approach to simulate smolt movement. The simulated individual smolts were allowed to navigate a simulated river section, with their movement rules based on statistically derived relationships between hydraulic variables and smolt movements past two hydropower stations.

The model aims to strike a balance between providing accurate predictions of smolt downstream movement in river sections past hydropower intakes while ensuring simplicity in model setup and parameterisation to facilitate further refinement and applicability across different systems. The model parameterisation and simulations were conducted in R^27^, version 4.3.1 and figures and maps was produced utilising the ggplot2 library^28^.

#### The river section

**Fig. 2:**
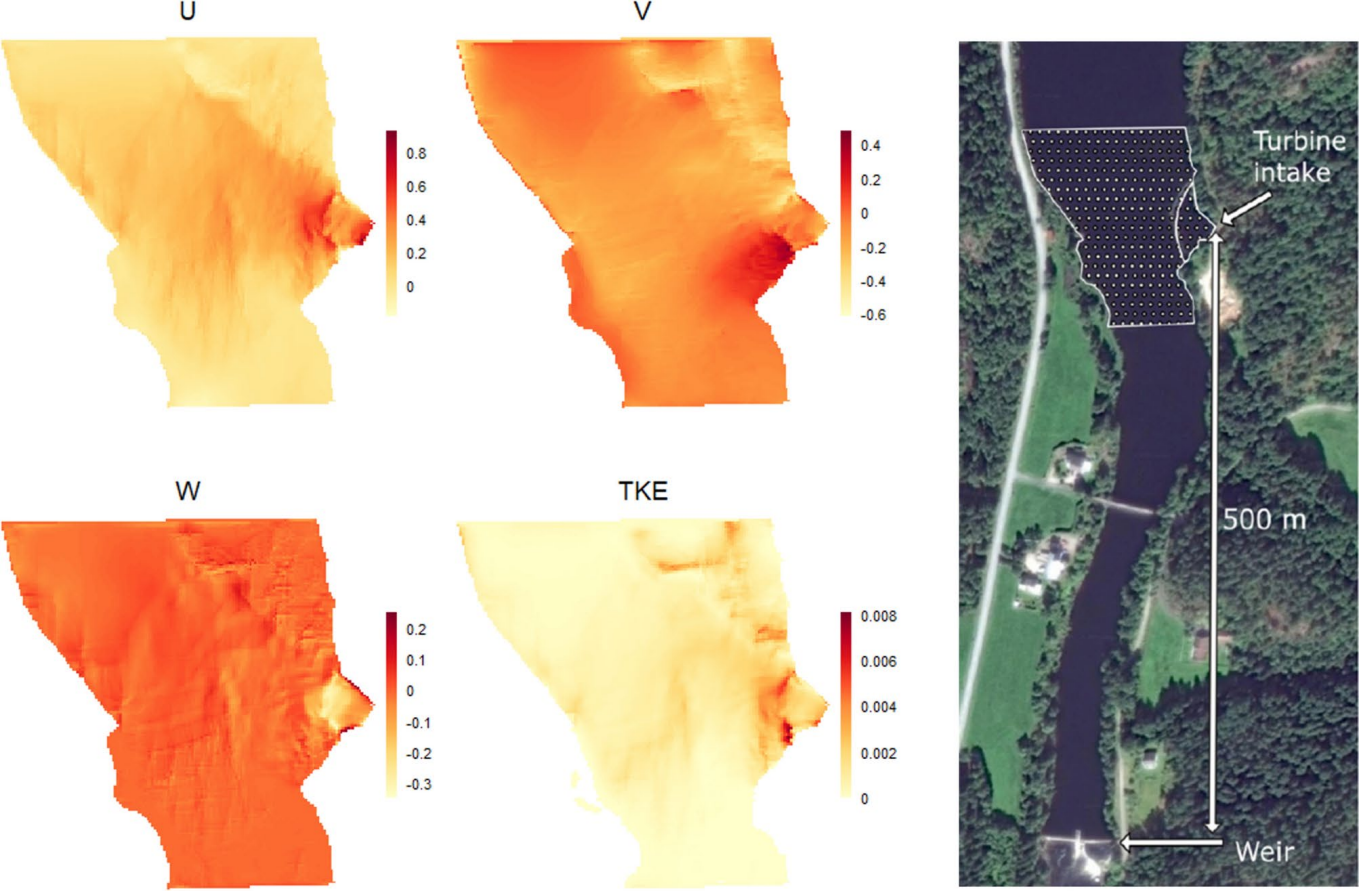
Rasterlayers for the diferent flow velocity components (u,v and w in m/s) and turbulent kinetic energy (TKE in m^2^/s^2^), the colors represent different values (legend) for the respective velocity components modeled which here are representative for the Q92 scenario within the river section in focus. The rightmost picture shows the section in the respective stream, with indications of where the turbine intake is situated. Image © 2024 Maxar Technologies.

The simulated river-section was defined by multiple cells (scaled from hexahedral cells with median length of 0.5 m in real river conditions, see Fig. 2), where the hydraulic condition within each cell was derived from the CFD-simulation scenarios described above. Consequently, the simulated river section mimicked natural static flow conditions for which CFD-simulations existed. Specifically, we utilised the R-library NetLogoR^29^ to set up the extent of the river section as a raster file, representing a user defined 2D horizontal plane of the water column. The second upper part (i.e., CFD-simulations for the second horizontal plane to the water surface, out of ten simulated horizontal planes of the water column) of the water column were chosen as a general starting point for all smolts, unless specifically altered for individual smolt simulations. The second upper layer was chosen as default as Atlantic salmon smolts commonly migrate in near-surface waters^21^. Each raster cell was assigned to specific hydraulic values of u, v, w and TKE, directly obtained from the output of CFD modelling (see Fig. 2). Whereas, for each cell the resultant velocity was derived by u-velocity and v-velocity through the utilization of the uv2ds-function within the rWind library^30^.

#### Movement model parameterization

Within our model setup, individual fish movements were analysed in a 2D horizontal plane for most of the river section, where small-scale movement decisions were functions of the varying hydraulic conditions within the section of interest. Reliable 3D movement data were only available in the immediate vicinity of the turbine intake (see Fig. 2), representing a small portion of the total dataset. Singular small-scale movements were defined as transitions between two cells in the simulation, corresponding to movements between patches approximately 0.5 m apart in real-world dimensions. The models were parameterised based on the relationships between observed smolt movements and estimated hydraulic variables (see “Biological and hydraulic data” above). From the data, we categorised each movement step to determine whether a fish was: 1) following the flow (+ /− 20°) or not; and, if not, 2) whether the fish was maintaining a consistent direction (+ /− 20°) deviating from the flow direction (directional swimming behaviour, e.g., swimming bursts) or frequently changing direction (non-directional swimming behaviour, e.g., searching). The tolerance of 20° was chosen to account for the uncertainty associated with the hydraulic models and the positional and directional observations of the fish at any given time. Furthermore, the 20° tolerance was not selected arbitrarily but was based on the distribution of angular differences between the resultant velocity and the calculated direction of the observed smolt within each cell. This distribution was highly left-skewed, with a high density of smolt directions within approximately 20° of the resultant velocity, and a long, more or less uniformly distributed tail from 30° to 180°.

We then constructed two models: 1) the “Follow-the-flow” model, which predicted whether the fish was likely to follow the flow or not, and 2) the “Swim-direction” model, which predicted whether, when not following the flow, the fish would randomly change direction or maintain the same direction as in the previous movement decision.

The parameter estimates for the two movement models were derived from boosted regression trees models (BRT)^31^, set up utilizing the dismo library^32^. To optimize the settings for the BRT (i.e. numbers of boosting trees, learning rate and tree complexity) we used a tenfold cross validation (see Elith et al. 2008). This process fits separate models of increasing complexity to 10 data subsets, each containing 70% of the data to assess the optimal number of boosting trees at a given learning rate and tree complexity. We run this cross-validation process for a range of combinations of learning rate (specifically: 0.01, 0.025, 0.005, 0.0025 and 0.001) and tree complexity (1 to 9), to find the most optimal setting giving best predictive performance based on predictive deviance and area under curve statistics^33^. The predictive deviance was defined as the unexplained variation when comparing observations against predictions of deviations from flow during the cross validation^31,34^. The final model used for predictions was then fitted to all observations using the optimal combination of numbers of boosting trees, learning rate and tree complexity as obtained from the validation procedure.

There are multiple alternatives to BRTs, but we chose BRT as the methods shows some predictive advantages over comparable modeling methods, has the flexibility to fit nonlinear responses while being relatively insensible to outlier, and select predictor variables and interactions among them, while calculating predictor relative importance. The modeling method could thus more easily be treated as a generic approach, accounting for variations in the data more easily compared to for example regression models, such as Generalized Additive Models (GAMs). However, the model setup does not consider potential hierarchical structure of individual fish movements (e.g., movement behavior might vary between individuals). Despite this limitation, we believe that movement probabilities for the average smolt are representative as we based these on a substantial dataset comprising 140 tagged smolts with 32,994 recorded movements across two distinct study systems (Orkla and Mandal).

BRT-models might also have a propensity to overfit models^35^, we mitigated this risk by exclusively incorporating variables previously demonstrated to influence smolt movement decisions in comparable studies (i.e., u, v, w, TKE)^13^, and also removing noisy data (e.g., obvious outliers in recorded tracks). It is important to note that our model exclusively considers observed movement trends as functions of hydraulic variables. In reality, however, smolts are likely influenced by a range of additional factors beyond hydraulics, including visual stimuli and various biotic and abiotic variables that are absent from our dataset. Consequently, addressing potential overfitting entirely may prove challenging without incorporating these additional variables, which were not available for this study.

#### Simulated individual smolts

**Fig. 3:**
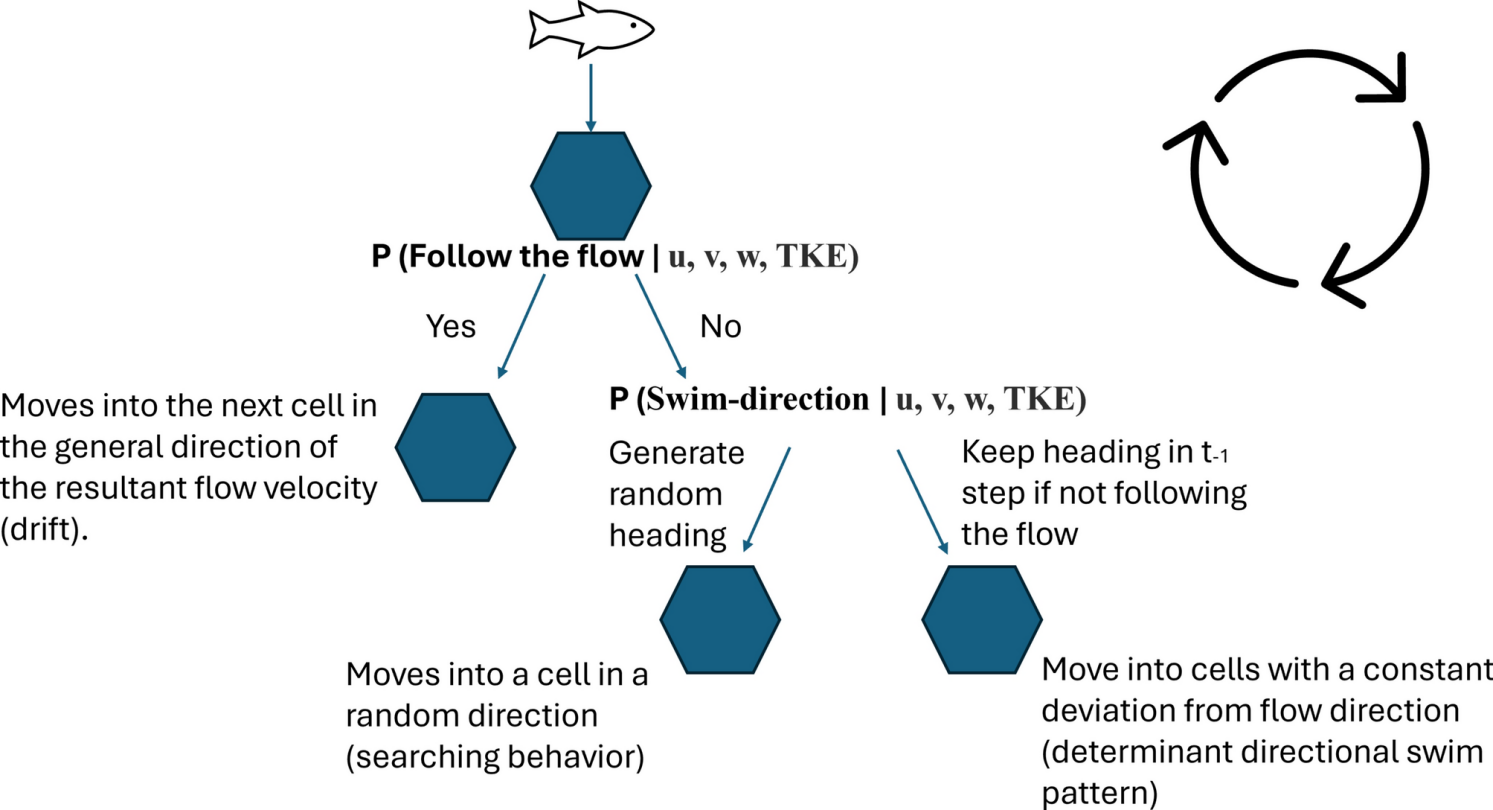
A conceptual figure of the flow diagram for the main movement rules in the IBM predicting the downstream migration paths for the smolts. A smolt enter a cell where the “Follow-the-flow”-model predicts the probability for the smolt to either follow the flow or not, based on the flow velocity components u, v and w and turbulence (TKE) in the specific cell. The probability is used in a binomial function determining the outcome. If the smolt do not follow the flow, then there is a second model predicting the probability whether the smolt should generate a random direction or keep the direction in the previous step (if not following the flow). The whole sequence was run in a loop until the smolt reaches its endpoint in the simulation.

Smolt simulations were undertaken for two different discharge (Q) scenarios, Q = 92 m^3^/s and Q = 98 m^3^/s). These scenarios were selected to closely mirror the hydrological conditions encountered by the majority of the studied smolts throughout their migration period, in the study conducted by Silva et al.^13^. For each discharge scenario, smolt movements were simulated from four different starting points, with five replicates per starting point. The four staring points were chosen to represent different entering points from left to right bank of the river section. The individual smolt (i.e., each replicate) was allowed to move one step at a time, from cell to cell (approx. 0.5 m in real life distance) in the simulated river section. When the smolt entered a cell, two movement probabilities were predicted, one from the “Follow-the-flow”-model and another from the “Swim direction”-model, based on the specific hydraulic variable values (u, v, w and TKE) in the cell. The predicted probability from the “Follow-the-flow” model was used as the probability in a binomial function to determine whether the smolt followed the main flow direction or not. If the smolt followed the flow, it was assigned a direction within 20° of the main flow direction and moved to the closest cell along that path. If the smolt was predicted not to follow the flow, the “Swim-direction” model provided probabilities for a binomial function to decide whether the smolt should adopt a new random direction (bounded between 0 and 360°, excluding the main flow direction ± 20°), or retain the heading assigned in the previous step. For the first instance in which the smolt was predicted not to follow the flow, a random direction was always generated. A conceptual flow diagram of the movement options can be seen in Fig. 3. These movement rules were executed in a loop until the smolt exited the boundaries of the river section, at which point it was removed from the simulation. The movement rules could be summarised into three main swimming behaviours influenced by hydraulic conditions: (1) following the main flow direction (drifting); (2) indeterminate deviation from the main flow direction (frequent directional changes within each cell, searching); and (3) determinate deviation from the main flow direction (maintaining a consistent deviation, exhibiting deterministic and prolonged directional movement away from the flow). All three behaviours mimicked the observed categorised behaviours in the data.

Essentially, the model functions as a spatially explicit movement model, wherein the hydraulic characteristics of each cell are defined by modelled discharge values for a preselected 2D plane of the water column.

#### Model performance and evaluation

To evaluate the model’s performance, in addition to assessing standard model metrics as outlined above, we simulated smolt movement under two distinct flow scenarios with representative conditions corresponding to those encountered by the observed smolts (Q92 and Q98). The simulated smolt trajectories were visually compared with observed movements under the same two discharge scenarios. To assess the model’s performance against a “drift only” scenario, we also generated simulations where the smolts exclusively followed the general flow direction (+ /− 20°) at each step. Additionally, we compared the expected fates (i.e., entering the turbine versus passing the weir) of the simulated smolts under different scenarios and settings against the fates of the observed smolts in the respective discharge scenarios.

To further evaluate the IBM’s ability to replicate specific individual tracks, we selected two observed tracks from each discharge scenario and simulated three smolts with initial conditions closely matching field conditions (i.e., similar starting points, track lengths, assumed swimming depths, and flow conditions). The four observed tracks were chosen based on their completeness, with as few missing segments as possible.

## Results

**Fig. 4:**
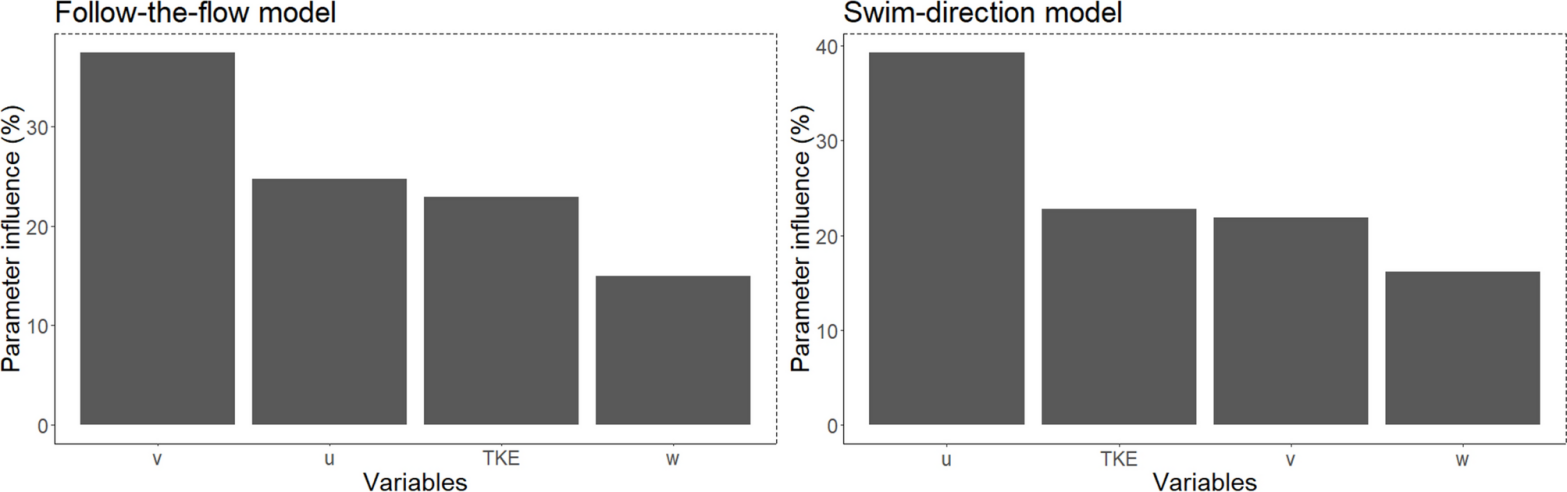
Parameter influence for the two BRT-models predicting probability of smolt movement decisions for whether a smolt follow the flow or not (left panel), and whether the smolt would obtain a searching behavior or a more deterministic directional swim pattern if not following the flow (right panel).

The two BRT models for smolt movement performed and generalised reasonably well, as indicated by high AUC values and relatively low deviance statistics (Table 1), given that the models predicted probabilities of movement based solely on hydraulic variables. There are, however, indications of moderate overfitting, as reflected by the CV (deviance) being 37.4% higher than the deviance measure (Table 1) for the “Follow-the-flow” model. For this model, the most influential variable was v (transversal velocity), followed by u (downstream/upstream velocity), TKE (turbulent kinetic energy) and w (vertical velocity), respectively (see Fig. 4). For the “Swim-direction”-model, the most influential variable was u, followed by TKE, v and w respectively (Fig. 4).

**Table 1:** Performance statistics for the two BRT-models predicting the probability for either following the flow or not (Follow-the-flow) and if not following the flow, change direction or keep previous heading (Swim-direction).

Model	Interaction depth	Shrinkage	Number of trees	AUC	CV (AUC)	Deviance	CV (Deviance)
Follow-the-flow	9	0.025	7000	0.934	0.850	0.697	0.958
Swim-direction	9	0.005	8600	0.857	0.801	0.744	0.829

Interaction depth, Shrinkage and Numbers of trees are all parameters that are used to optimize the BRT. AUC (Area Under the Curve) is used to evaluate how well the model discriminates. AUC values range from 0.5 (no better than random guessing) to 1 (perfect classification). A small difference between training AUC and cross-validated CV (AUC) indicates a well-generalized model. Deviance is a measure of how well the model’s predicted probabilities match the actual labels. A small difference between the training deviance and CV (Deviance) suggests the model is fitting well and generalizing appropriately. A large difference points to overfitting.

The simulation of smolts movements past the river section was relatively fast. A simulation of 1000 smolts that was allowed to move a maximum of 1000 steps each, took approximately 1 h and 15 min on a normal, but aging Dell laptop (Intel Core i7-8650U CPU: 1.90 GHz—2.11 GHz, 16 GB RAM). This could be significantly faster using a faster computer, and or utilizing parallel computing.

**Fig. 5:**
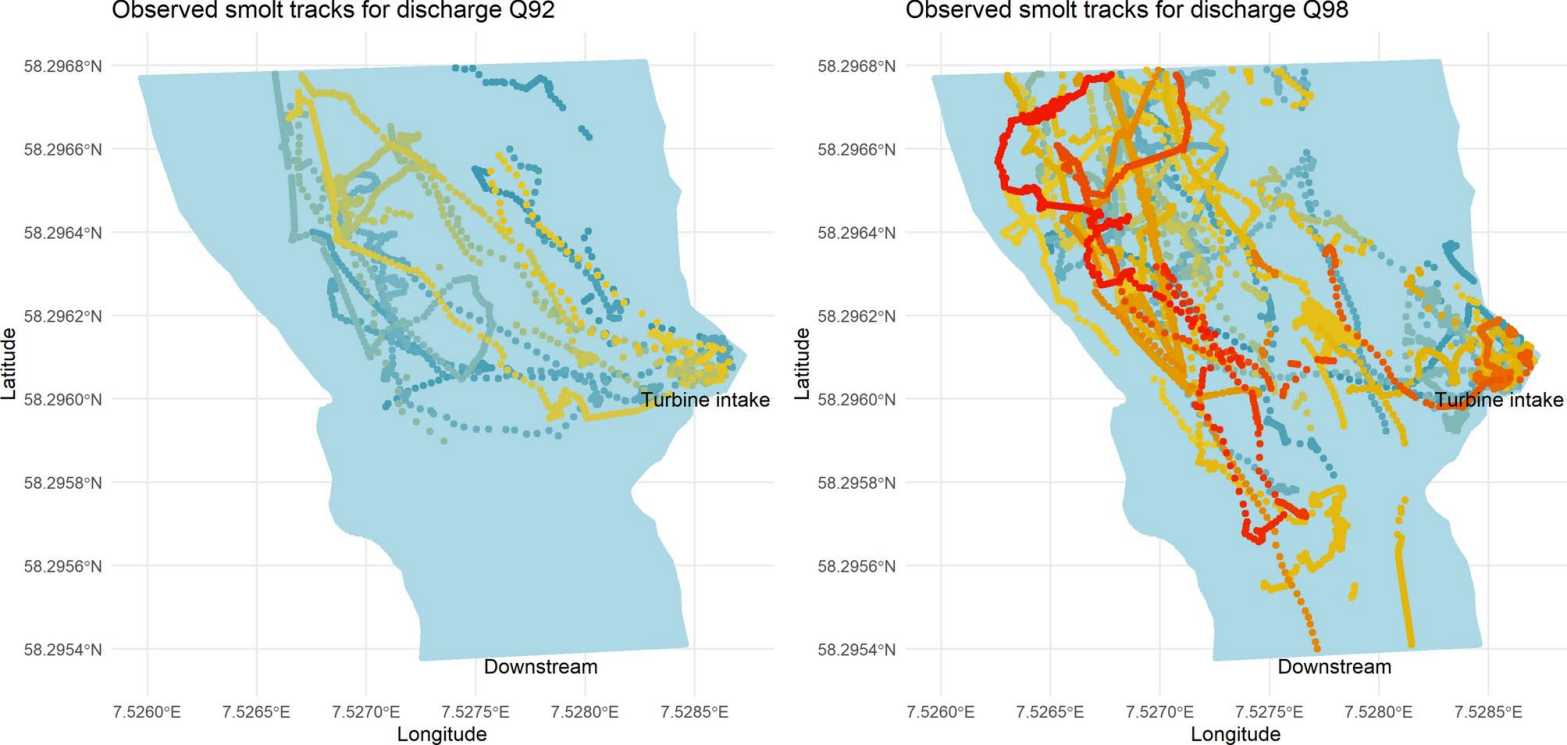
Observed smolt tracks under two different discharge scenarios (Q92, left panel and Q98, right panel). The different colors of the tracks represent different individual smolts.

**Fig. 6:**
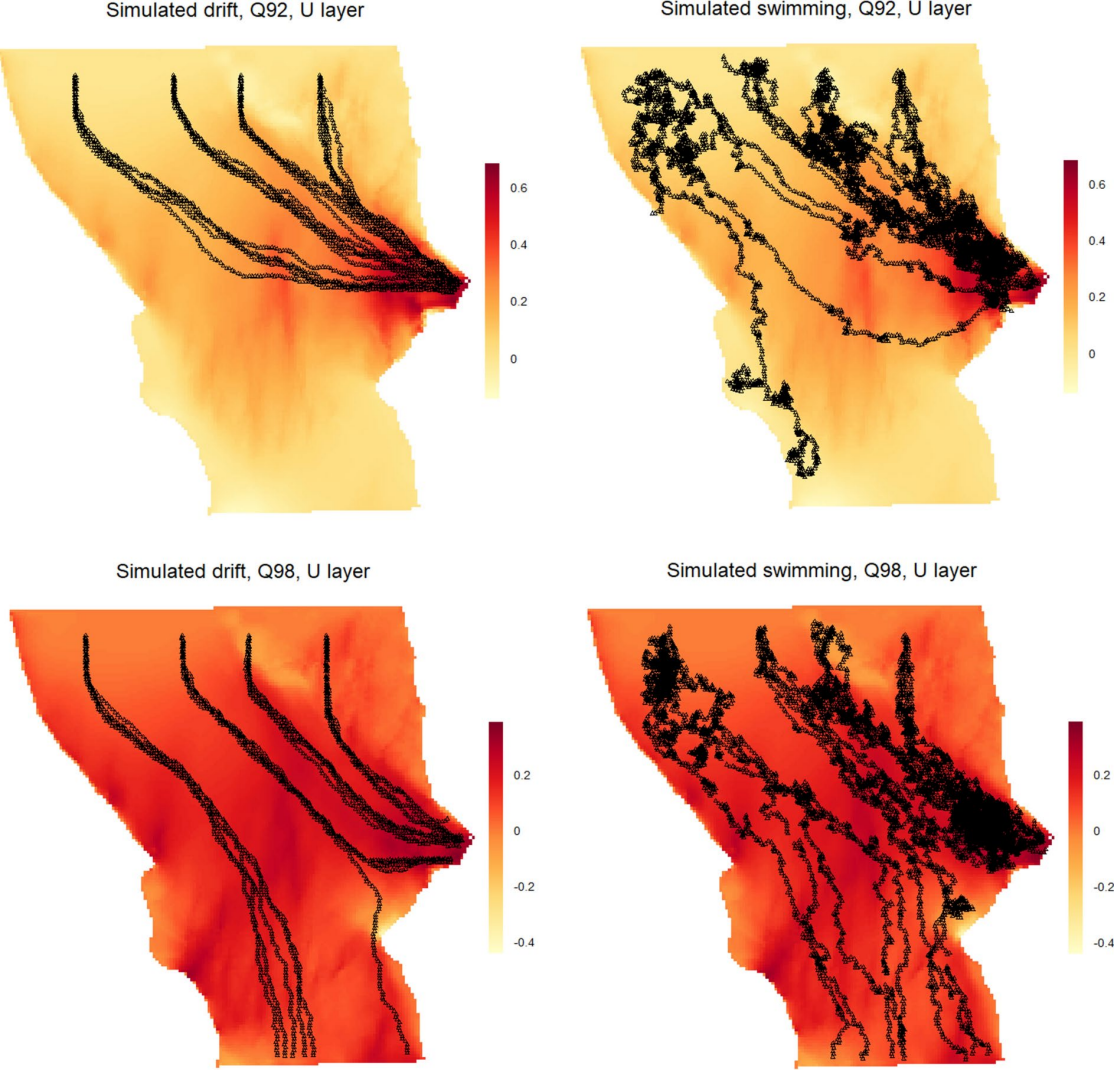
Simulated smolt tracks under two different discharge scenarios (Q92 and Q98), based on the constructed IBM for a random drift setting (smolts following the flow, left) and smolts that are allowed to actively alter their swimming behavior as a function of the hydraulic environment (right). The colors represent different velocities for the u layer (downstream/upstream) for the second uppermost vertical plane (out of ten simulated in the water column), and the scales of the values are discharge scenario specific. For the four simulations we chose four common starting spots, where five smolts were released at each spot.

**Fig. 7:**
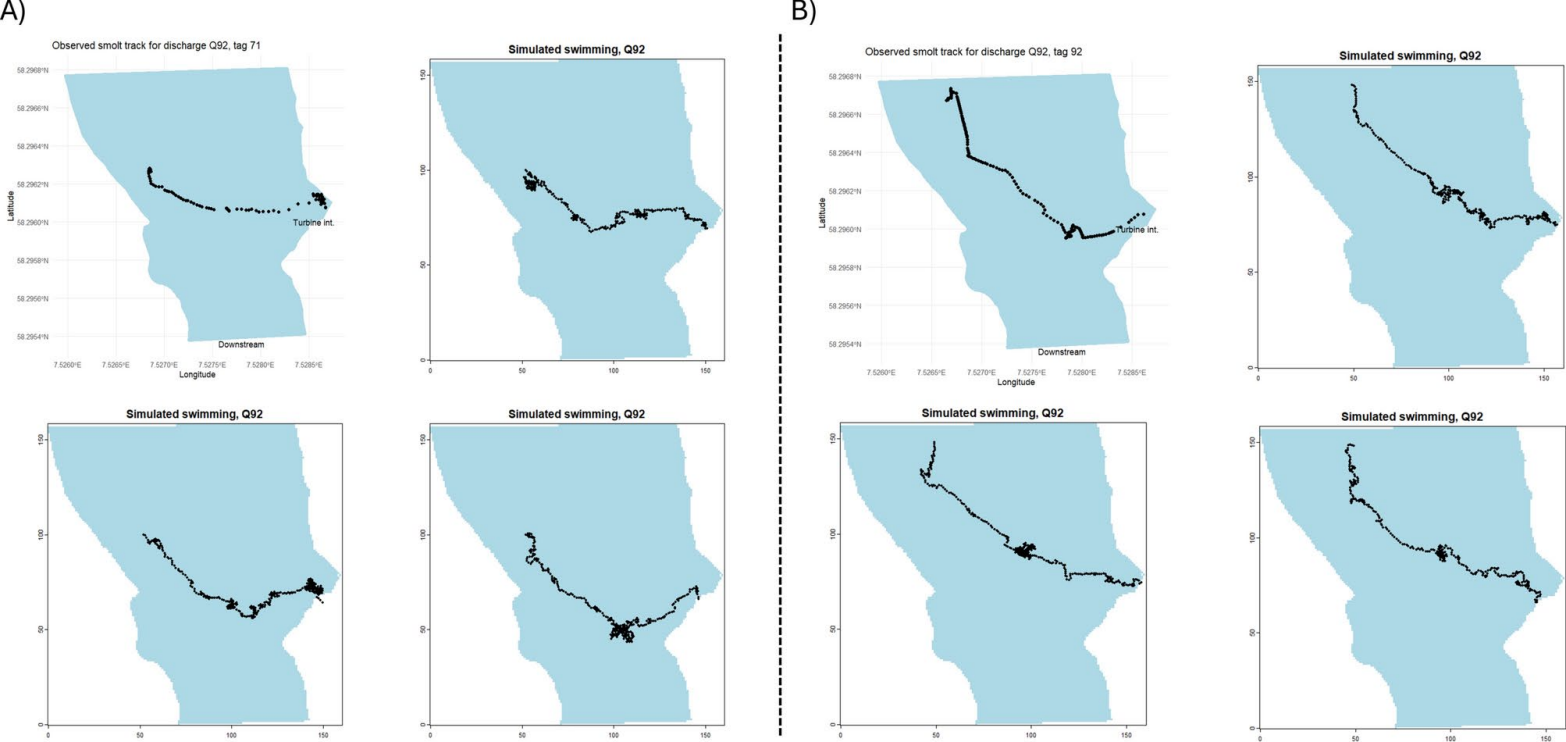
Observed tracks (top left panel) for two individual smolts (a and b) passing the river section during the Q92 discharge scenarios. The three other panels in a and b represents associated individual smolt simulations with initial conditions that closely match field conditions (i.e., similar starting points, number of points in track, assumed swimming depth and flow conditions).

**Fig. 8:**
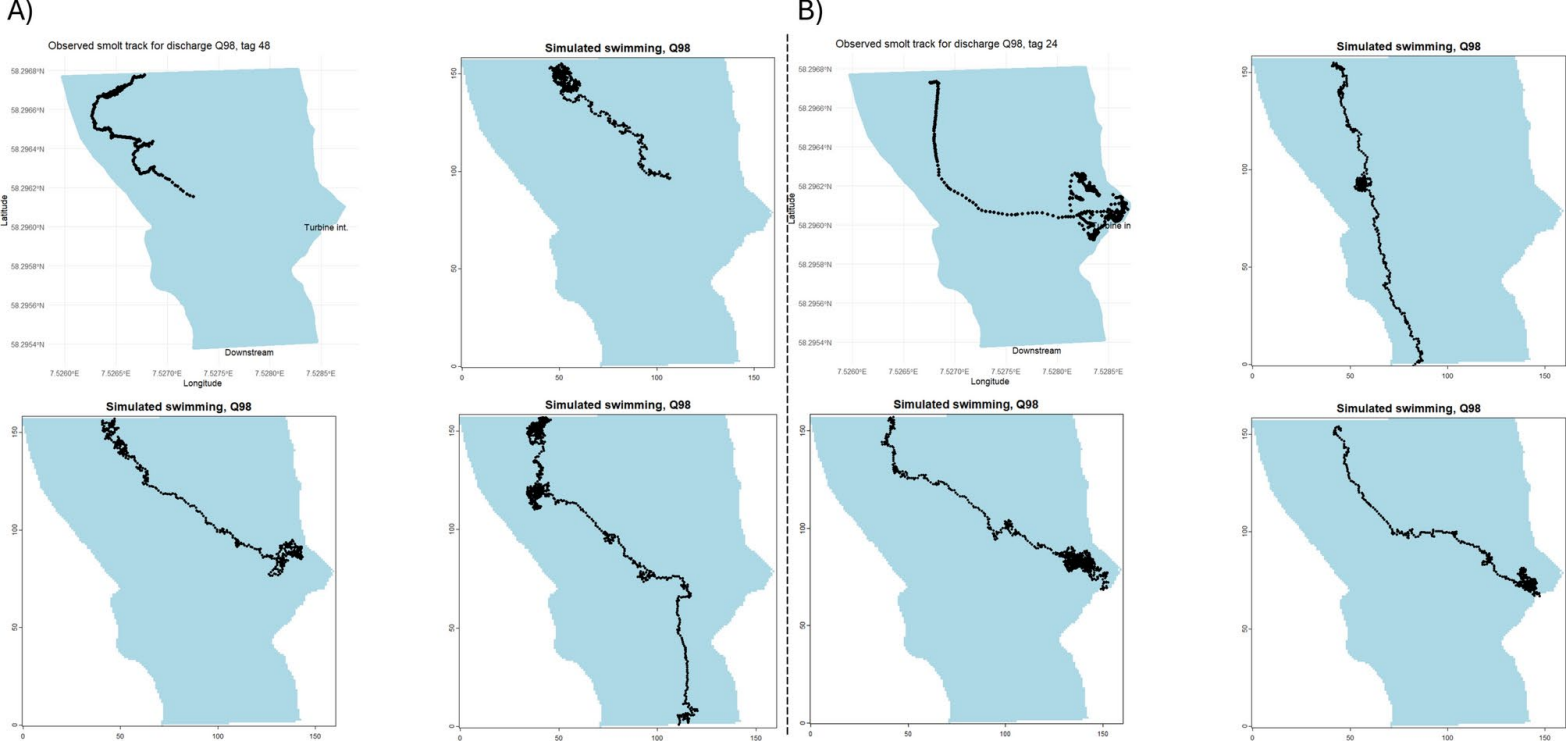
Observed tracks (top left panel) for two individual smolts (**a** and **b**) passing the river section during the Q98 discharge scenarios. The three other panels in a and b represents associated individual smolt simulations with initial conditions that closely match field conditions (i.e., similar starting points, number of points in track, assumed swimming depth and flow conditions).

Overall, the IBM produced simulated smolt tracks that were comparable to the observed tracks (Figs. 5, 6, 7 and 8). The IBM simulations outperformed the “drift only” simulations in representing observed swimming behaviour, as the observed smolt tracks generally exhibited far greater complexity than those produced by the “drift only” model.

When visually comparing specific observed individual tracks against simulations with closely matching settings, the IBM was able to replicate the swimming behaviour, both in terms of swim trajectory and the complexity of the trajectory (Figs. 7 and 8). However, there were some notable variations in the simulations for the individuals under the Q98 discharge scenarios. In these cases, the IBM successfully captured and mimicked areas where the observed smolts exhibited signs of searching behaviour (e.g., multiple points in the same area and tracks going back and forth), although the outcomes of the simulations varied (Fig. 8). There was also a tendency for the IBM to simulate “searching behaviour” (i.e., generating random headings for the smolts) more often than was observed in many of the actual smolt tracks, although this varied significantly between individual tracks (see Fig. 5). Comparing the expected fate (i.e., going into the intake or passing) of the simulated smolts against the observed tracks revealed no distinct differences between the scenarios, except for a slightly higher likelihood of bypassing the turbine intake area in both the observed and the simulated swimming behaviour simulations, compared to the drift-only simulation (10 smolts bypassed when simulating swimming behaviour and 6 smolts bypassed during drift).

## Discussion

Our model framework was able to produce representative migration patterns for smolts under various flow conditions in a river section past a hydropower intake, utilising predictive models based on statistical relationships between swimming behaviour and hydraulic variables. The correlative functions within the individual-based model (IBM) appear to capture the general movement patterns of smolts under the flow velocities for which they were parameterised. The IBM outperformed the drift-only simulation in providing representative smolt tracks. However, the IBM tended to overestimate the likelihood of smolts exhibiting prolonged searching behaviour in certain areas before selecting and maintaining a new steady heading. This tendency became especially evident when visually comparing specific observed tracks with simulations under similar conditions. Although the excessive searching behaviour in the simulations usually did not significantly alter the overall movement trajectories, it did result in longer simulation times for smolts in some areas. Notably, the areas where searching behaviour occurred in the simulations corresponded well with observed searching behaviour in individual tracks (areas with numerous recorded points or back-and-forth movements). There were some intriguing instances where simulations with similar settings (e.g., similar starting points) produced differing outcomes for this searching behaviour (see Fig. 8). Interestingly, these were also areas where observed tracks with approximately the same starting points diverged under the same discharge scenarios. Thus, areas in the IBM simulations that exhibited excessive searching behaviour may indicate “problematic” flow velocities and highlight focal zones for mitigation efforts (e.g., structural adjustments to modify flow velocities), to more effectively guide the smolts past these zones and the hydropower intake.

Fish decision-making is a complex process influenced by acute and non-acute responses. Acute responses, such as avoiding predators or escaping dangerous hydraulic conditions, are more consistent across contexts, while non-acute responses depend on competing stimuli^36^. The constructed IBM used in this study obviously represent simplifications of the environment and fish movements and does not include the range of biotic and abiotic factors that might also influence swimming behavior of fish^37^. In our model, the variation introduced by unaccounted factors was treated as noise or attributed to hydraulic variables, which may have contributed to the observed overfitting and an overemphasis on the measured variables. Despite this, the model performed well in capturing the complex, chaotic, and natural movement patterns of smolts within the system. However, due to signs of moderate overfitting in one of the models within the IBM, it does not necessarily produce reliable simulations beyond the data on which it was parameterised.

The decision to exclude detailed biotic factors was intentional, given the difficulty in quantifying them and in favour for faster model performance. However, future models should strive to incorporate such site-specific influences for greater accuracy. For instance, research has shown that Atlantic salmon smolts do not always follow the main flow direction near hydropower intakes, suggesting that movement decisions in these areas may be more influenced by abiotic factors beyond hydrological variables (e.g., noise, physical barriers such as racks that force the smolts to dive, etc.).^38,39^. While our current model provides a generalised approach that can be refitted and applied to different systems, this may come at the cost of reduced precision near hydropower intakes. Nonetheless, the probabilistic movement decisions allow users to explicitly account for this uncertainty if these variables are measured and included in future iterations. It is also worth noting that, even when based solely on hydraulic variables, the current model parameterisation appears to better capture the more chaotic movement patterns observed in front of the intake compared to a “drift-only” simulation.

Another limitation of the current model parameterisation is its reliance on data with the assumption of a constant depth interval for most tracks, due to the lack of reliable smolt depth data across the river section. Consequently, the current IBM simulations also assume a constant predefined depth that the simulated smolts follow. Although variable depth for each cell in the simulation could be technically implemented, this would require a finer resolution of observed smolt tracks in the 3D environment throughout the river section to be more reliable than the current assumption of constant depth. While the assumption of near-surface migration can be justified for most of the riverine journey of smolts, evidence in the literature suggests that smolts sometimes remain in the deepest part of the water column, likely as an anti-predator behaviour.^40,41^.

Further development of the IBM should include higher-resolution tracking data from different sites to test the consistency of trends in swimming behaviour as a function of measured flow velocities and TKE, across a broader spectrum of topographic and geometric features. As mentioned above, more research is also needed to better understand and capture the utilisation of different depths as functions of multiple biotic and abiotic variables. For example, what is the functional relationship between depth use during migration and the density of a specific predator, such as pike (*Esox lucius*)? Understanding and incorporating relationships between swimming behaviour and the suite of biotic and abiotic variables within the IBM framework is a natural next step. Including additional variables, and parameterising and testing the model across more sites, is likely to reduce the moderate overfitting observed in one of the models (thereby reducing the noise in the data) and enhance both the robustness of the model and the reliability of its predictions.

Additionally, since this model was developed using tracking data from Atlantic salmon smolts, which also were similar in size, and fish behavior is highly species- and life-stage/size-specific due to the varying biomechanical and physiological characteristics of individuals^42^, the model’s application is limited. Therefore, there is a need for future development of this and similar models focusing on other fish species, sizes and life stages.

## Conclusions

In a heavily influenced natural world, managing nature is often a trade-off between maximizing benefits for humans (for example energy production) and minimizing ecological stressors. To reduce the ecological stressors of hydropower following an adaptive management framework, it is essential to have model concepts that can predict the ecological outcome of different hydropower operations before they are implemented. This allows for an adaptive process before costly and potentially ineffective measures are constructed. Our model seems to adequately capture and predict general pattern of smolts movements through a river section using the understanding of how the smolt might change their behavior according to the hydraulic environment, which seems to be comparable across river systems and populations^20^. Thus, as our model are easy to reparametrize for different settings and areas, and produces output relatively fast, we believe the model concept to be a valuable tool for exploring potential effects on smolt migration patterns based on solutions that will change the hydraulic properties of the river section. However, the generality and the simplicity of the model comes at the cost of reduced precision of the individual track. This might not necessarily be a big concern, if the goal is to explore general population effects as a result of multiple mitigation solutions before implementation.

## Data Availability

The data and code presented in this study are available on request from the corresponding author.
